# Unlocking seagrass germination: divergent roles of strigolactones and smoke-water in *Zostera marina* (Zosteraceae)

**DOI:** 10.3389/fpls.2025.1629832

**Published:** 2025-11-18

**Authors:** Riccardo Pieraccini, Lisa Picatto, Nico Koedam, Ann Vanreusel, Tobias Dolch, Tom Van der Stocken

**Affiliations:** 1 Marine Biology Research Group, Department of Biology, Ghent University, Ghent, Belgium; 2 Ecology, Evolution and Genetics Research Group (bDIV), Biology Department, Vrije Universiteit Brussel, Brussels, Belgium; 3 The Joint Programming Initiative Healthy and Productive Seas and Oceans (JPI Oceans), Brussels, Belgium; 4 Coastal Ecology, Alfred-Wegener-Institut Helmholtz-Zentrum für Polar- und Meeresforschung – Wattenmeerstation Sylt, List, Germany

**Keywords:** seagrass, seed germination, smoke-water, strigolactones, karrikins, plant growth regulators, dormancy regulation, seagrass restoration

## Abstract

Seagrasses, such as *Zostera marina*, play a crucial role in coastal ecosystems, yet the hormonal regulation of their seed dormancy and germination remains poorly understood. Strigolactones (SL) and karrikins (KAR), two plant growth regulators (PGRs) known to regulate germination and development in terrestrial plants, have recently been identified in marine angiosperms. However, their functional roles in seagrasses remain unexplored. Here, we provide the first assessment of SL and smoke-water effects on *Z. marina* seed germination and seedling development under controlled conditions. Smoke-water is a solution derived from plant combustion that contains a complex mix of bioactive compounds, rich in butenolide compounds such as karrikinolides (e.g. KAR_1_, KAR_2_). We tested the effect of ten different concentrations of SL and smoke-water on germination percentage, mean germination time, and seedling growth, considering multiple seed generations. SL significantly promoted germination, particularly at intermediate concentrations (3–15 mg L^−1^), where germination percentages reached up to 46.7%, as compared to 16.4% in the controls. In contrast, smoke-water treatments reduced germination to below 5% across all tested dilutions, delaying or preventing germination. Moreover, SL enhanced cotyledon growth and accelerated germination, whereas smoke-water consistently inhibited early seedling development. The identification of *Z. marina* orthologs of key SL and KAR signaling components suggests evolutionary conservation of these pathways in marine plants. Our findings provide new insights into the hormonal regulation of seagrass germination, highlighting both conserved and divergent functions of SL and KAR compared to terrestrial species. These results advance our understanding of hormonal control in marine plant species and hold implications for the conservation and restoration of seagrass meadows.

## 1 Introduction

Marine plants exhibit a diverse range of physiological and morphological adaptations that enable them to thrive in highly variable and dynamic coastal ecosystems (Touchette, 2007; Beer et al., 2014; Reusch, 2014). These adaptations include salinity tolerance (Nejrup and Pedersen, 2008), responses to temperature fluctuations (Lee et al., 2007), and mechanisms to cope with light limitation (Beer et al., 2014). Beyond these well-studied traits, metabolic and hormonal pathways regulating development, growth, root architecture, and seed dormancy are also essential for plant population resilience in dynamic marine habitats (Pieraccini et al., 2025). Among marine angiosperms, adaptation to permanently submerged and saline environments has been accompanied by distinct evolutionary changes, including the loss of genes associated with stomatal function and ethylene signaling, as observed in *Zostera marina* (Yuan et al., 2019; Zhang et al., 2022). Seed dormancy represents a key adaptive trait contributing to the long-term persistence of seagrass populations (Halliday and Fankhauser, 2003; Shu et al., 2016), enabling seeds to endure unfavorable conditions and synchronize germination and seedling emergence with optimal seasonal cues (Orth et al., 2000; Yang et al., 2019).

Dormancy regulation in plants involves complex hormonal signaling, particularly the interaction between abscisic acid (ABA) and gibberellins (GA), which together modulate the transition from dormancy to germination (Shu et al., 2016; Liu and Hou, 2018). This interplay is also believed to operate in *Z. marina*, where similar physiological patterns of dormancy and germination have been observed (Orth et al., 2000; Pieraccini et al., 2025).

Dormancy is especially important for species like *Z. marina*, where sexual reproduction is key to population persistence and meadow expansion in dynamic coastal ecosystems (Harrison, 1991; Potouroglou et al., 2014). In coastal and intertidal ecosystems, *Z. marina* populations experience substantial environmental fluctuations, including daily, tidally driven variations in temperature and salinity, as well as seasonal and climate change-induced shifts. These conditions make dormancy an important ecological strategy (Orth et al., 2000). Under such conditions, dormancy functions as an ecological buffer, allowing seeds to remain viable until environmental cues signal favorable conditions for germination and growth (Statton et al., 2017; Pieraccini et al., 2025). Endogenous modulation of germination timing enhances the likelihood of successful establishment under optimal conditions (Brenchley and Probert, 1998; Touchette, 2007). This mechanism parallels strategies observed in desert ephemerophytes, where seed dormancy ensures emergence during brief wet periods, enabling survival in harsh climates (Finch-Savage and Leubner-Metzger, 2006; Klupczyńska and Pawłowski, 2021).

Although the ecological significance of dormancy is well established, the molecular mechanisms underlying its regulation in marine plants remain poorly understood. In terrestrial species, ABA and GA are established regulators of seed dormancy, while emerging evidence highlights the involvement of other plant growth regulators (PGRs), including strigolactones (SL) and karrikins (KAR), in the germination processes (De Cuyper et al., 2017; Brun et al., 2018). However, their roles in marine angiosperm, including *Z. marina*, remain unexplored.

Strigolactones (SL), first identified in cotton root exudates and named after the parasitic genus *Striga* (Xie et al., 2010), are host-derived signaling molecules that act across species boundaries, stimulating seed germination in certain parasitic plants (Matusova et al., 2005). Beyond their role in interspecies communication, SLs regulate a wide range of developmental processes, including shoot branching, root architecture, and symbiosis with mycorrhizal fungi (Waters et al., 2017). Although the specific source and function of SLs in *Z. marina* have yet to be confirmed, their widespread occurrence across angiosperms suggests that similar signaling mechanisms could operate in marine environments. regulating shoot branching, modifying root architecture, and mediating symbiosis with fungi (Waters et al., 2017). KAR, which share structural similarities with SL, are combustion plant-derived butenolides that promote seed germination, photomorphogenesis, and stress tolerance in terrestrial plants (Flematti et al., 2004; Chiwocha et al., 2009; Nelson et al., 2012). Smoke-water, a natural source of karrikin activity (Pandey et al., 2025), contains a mixture of combustion-derived compounds, including karrikinolide (KAR_1_) and related molecules that act as germination cues in fire-adapted terrestrial plants (e.g. *Pinus* spp.) (Flematti et al., 2004; Chiwocha et al., 2009; Light et al., 2009; Jamil et al., 2014). Both SL and KAR pathways converge on the F-box protein MORE AXILLARY GROWTH 2 (*MAX2*), regulating developmental processes through the ubiquitination of SUPPRESSOR OF *MAX2*-LIKE (*SMXL*) proteins (De Cuyper et al., 2017; Zhang et al., 2019).

Recent genomic studies have identified *MAX2* and other signaling components in *Z. marina*, suggesting that similar regulatory mechanisms may operate in seagrasses (Olsen et al., 2016; Ma et al., 2024). Yet, despite these molecular similarities, their physiological roles remain unknown. To date, no study has examined the potential effects of SL and KAR on germination and early seedling development in *Z. marina*, representing an important knowledge gap. Understanding whether SL and KAR signaling influences germination and early seedling development in *Z. marina* could provide insight into the evolutionary conservation of hormonal mechanisms and their ecological relevance in marine environments.

This study aimed to assess the effects of SL and smoke-water on *Z. marina* germination and seedling development. We tested ten concentrations of each treatment across multiple seed generations under controlled *in vitro* conditions to determine whether SL and smoke-water act as germination inducers and to explore potential functional similarities between terrestrial and coastal plant systems, thereby assessing whether hormonal mechanisms known from land plants are conserved in marine environments. Although these environments and their species are geographically distinct, connections via rivers and lagoons suggest the possibility of shared or conserved plant regulatory mechanisms across the land–ocean continuum. Our findings provide a first step toward understanding how hormonal regulation shapes seed physiology in marine angiosperms and may inform strategies to improve seed-based seagrass restoration.

## 2 Materials and methods

### 2.1 Identification of biosynthesis and signalling genes in *Zostera marina*

Major gene families associated with the biosynthesis and signaling pathways of the PGRs strigolactones (SL) and karrikins (KAR) in *Z. marina* were retrieved from Olsen et al. (2016). Orthologous of relevant genes were compared with those in the model species *Arabidopsis thaliana* and analyzed using The Arabidopsis Information Resource (TAIR; https://www.arabidopsis.org). The TAIR ‘Plant Orthologs’ tool was employed to screen the annotated *Z. marina* genome for orthologs genes in this species.

This analysis aimed to confirm the presence of these SL- and KAR-related genes in *Z. marina* and assess the potential applicability of PGR-based treatments in this species.

### 2.2 Sample collection, treatment and storage

Reproductive shoots bearing seeds of the seagrass *Z. marina* were manually collected during low tide from Hamburger Hallig, Wadden Sea, Germany (54°35’52.3” N, 8°48’47.3” E) in late August of 2021 and 2022. Sampling permits were granted by Landesbetrieb für Küstenschutz, Nationalpark und Meeresschutz Schleswig-Holstein (Germany), on July 25^th^, 2021 and on August 1^st^, 2022. Collected shoots were transported to Ghent University, Belgium, in a refrigerated car cooler and maintained at 10 ± 1°C, for a total of 12 hours. Upon arrival, shoots were stored in 30 L barrels filled with natural seawater (36 PSU) under constant darkness at 10 ± 1°C for 45 days to allow natural seed release. Gentle water agitation facilitated release, and metal grids prevented debris accumulation while allowing seeds to settle at the bottom.

Following release, seeds were stored at 4 ± 1°C in darkness to mimic winter conditions and dormancy at the sampling site (cold stratification). During storage, seeds were treated with copper sulfate (CuSO_4_; Sigma-Aldrich) to prevent infections by oomycetes *Phytophthora gemini* and *Halophytophthora* sp (Govers et al., 2017). They were kept under gentle agitation in a 0.2 mg L^-1^ CuSO_4_ solution prepared in natural seawater, refreshed weekly. Seeds collected in 2021 (generation 2021) were stored for 15 months to evaluate the effects of prolonged dormancy, while seeds from 2022 (generation 2022) were stored for 4 months, representing natural overwintering duration at the sampling site.

**Figure 1:**
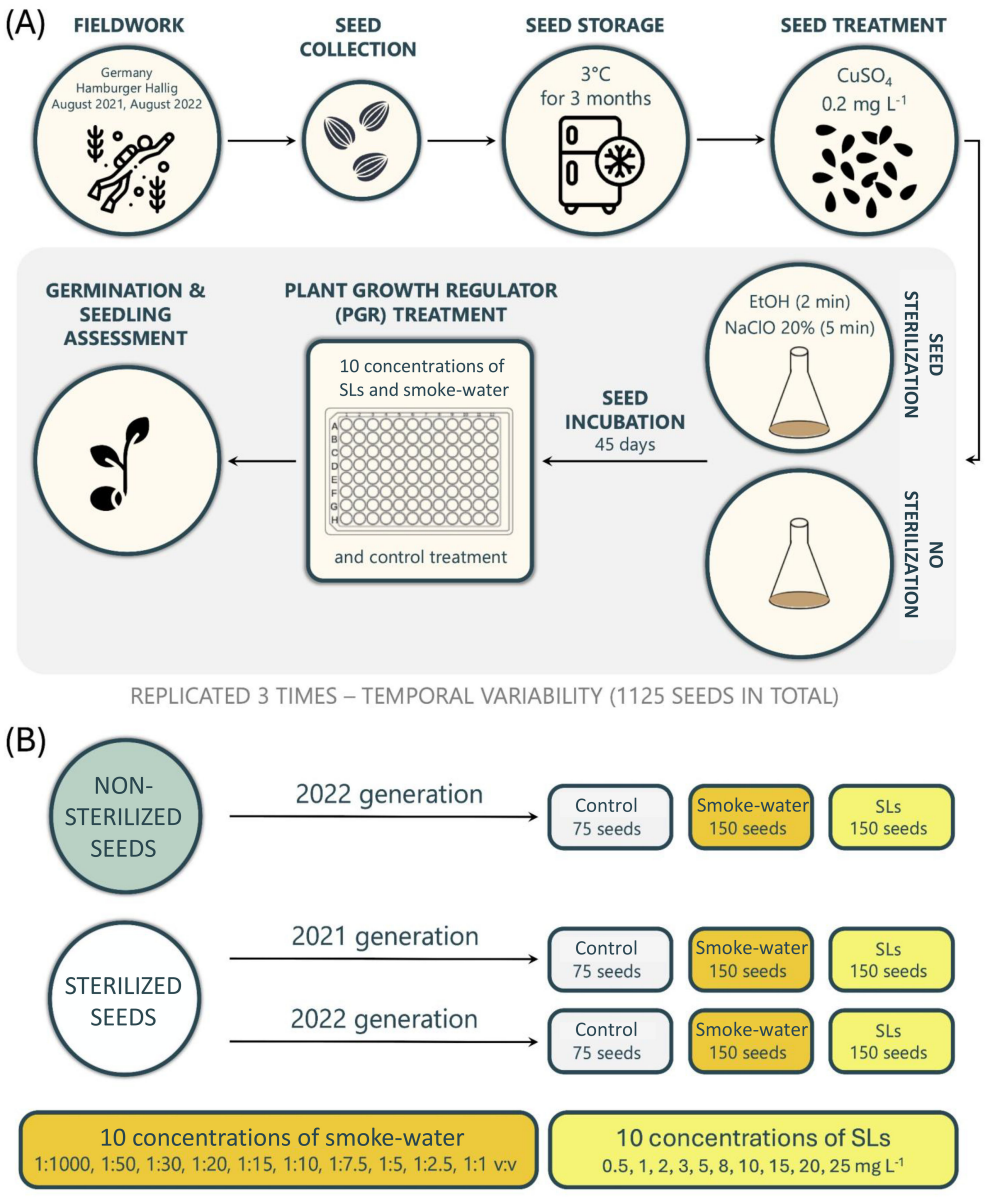
**(A)** Schematic representation of the experimental workflow, from field collection to experimental monitoring; **(B)** Schematic representation of the experimental design.

Before germination assays, seeds from generation 2021 and a subset of seeds from generation 2022 were surface sterilized first treated with 70% v/v ethanol (EtOH; Chem Lab NV) for 2 minutes and then with 5% sodium hypochlorite (NaClO; Sigma-Aldrich) in sterilized adapted seawater (30 PSU, hereafter SSW) for 20 minute and rinsed five times with SSW. SSW was prepared by adjusting the natural seawater salinity to 30 PSU with Milli-Q water and autoclaving the solution (121°C, 15 psi, 20 min). A seed sterilization protocol was adopted to reduce bacterial and algal growth and increase the efficacy of the PGRs solutions during the experiment. EtOH and NaClO are common chemicals used during *in vitro* culture to reduce contamination. For seed generation 2022, a full batch of non-sterilized seeds (NS 2022) was used as the control. For this generation, both PGR treatments (strigolactones and karrikins) and their respective concentrations were applied and adopted as the reference group. An overview of seed generations, treatments, and sample sizes is provided in **Figure 1**.

### 2.3 Plant growth regulator solution preparation

Strigolactones (SL) solutions were prepared by dissolving the synthetic analog GR24 (Merck) in sterile seawater (SSW) at the following concentrations: 0.5 mg L^-1^, 1 mg L^-1^, 2 mg L^-1^, 3 mg L^-1^, 5 mg L^-1^, 8 mg L^-1^, 10 mg L^-1^, 15 mg L^-1^, 20 mg L^-1^ and 25 mg L^-1^. The concentrations adopted in this study are consistent with those reported in prior research on seed germination, particularly in parasitic species such as *Striga* and *Orobanche* (Pouvreau et al., 2013; Chen et al., 2021).The smoke-water solution (KAR-rich stock) was prepared according to the protocol described by Coons et al. (2014). Smoke was generated by the combustion of 100 g of a herbal and straw mixture for 60 minutes (Original Ludwigs). The smoke was channeled into a flask to allow the water-soluble compounds to dissolve. The resulting smoke-water solution was then filtered through Whatman Grade 6 paper to remove larger particles. A series of ten KAR concentrations were prepared by diluting the smoke-water stock solution at the following ratios: 1:100 v:v, 1:50 v:v, 1:30 v:v, 1:20 v:v, 1:15 v:v, 1:10 v:v, 1:7.5 v:v, 1:5 v:v, 1:2.5 v:v, and 1:1 v:v. To ensure sterility, the solutions were further filtered through 0.2 µm syringe filters (Pall, Acrodisc) under sterile conditions in a laminar flow hood, preventing from contamination (Aslam et al., 2014).

The herbal mixture used to generate smoke-water (Original Ludwigs^®^ herbal and straw blend, Germany) was deliberately selected for its low lignin content, as such mixtures are known to produce fewer inhibitory phenolic compounds during combustion (Light et al., 2009, 2010; Kamran et al., 2017). Smoke-water can contain a variety of organic compounds derived from the combustion of plant materials, such as butenolides (karrikins), cyanohydrins, and hydroquinones. These compounds can have varying effects on seed germination; while butenolides are known to stimulate germination (Flematti et al., 2004), hydroquinones and cyanohydrins can alter germination outcomes (Light et al., 2009, 2010; Kamran et al., 2017). By selecting a herbal mixture with very low lignin content, we aimed to circumvent the formation of inhibitory compounds during combustion while maximizing the concentration of germination-promoting butenolides in the smoke-water solution. Smoke-water was prepared in-house, as pure karrikinolides (e.g., KAR_1_, KAR_2_) were not commercially available in Belgium at the time of the study. Moreover, smoke-water provides a more ecologically relevant mixture, since karrikins and related compounds naturally occur only as combustion by-products rather than as isolated molecules.

### 2.4 Experimental design and monitoring

A total of 1125 seeds were used in this experiment, encompassing three seed generations or treatments: sterilized 2022 (S 2022), non-sterilized 2022 (NS 2022), and sterilized 2021 (S 2021). For each generation, 150 seeds were assigned to the strigolactone (SL) treatment and 150 seeds to the smoke-water treatment, each tested across ten concentrations. An additional 75 seeds per generation served as controls, receiving no plant growth regulators (PGRs). This design yielded 900 treated seeds (450 per PGR) and 225 control seeds, totaling 1125 seeds overall (**Figure 1**). To account for temporal variability, the experiment was conducted in three independent trials, spaced two weeks apart, collectively encompassing the full set of 1125 seeds. Within each trial, each concentration and PGR treatment was replicated five times, ensuring experimental consistency and reproducibility.

#### 2.4.1 Seed incubation

Seeds were randomly assigned to treatments and placed individually in the wells of 96-well plates, with each well filled with 100 µL of hormone solution or SSW for control. Each hormone concentration had 15 replicates, 5 replicates within the well-plates and 3 between plates. Plates were incubated at 10 ± 1°C with a 16:8h light-dark photoperiod using full spectrum lights (T8 2FT LED grow light BL-D60A, Wolezek) at 100-120 µmol m^-^² s^-1^ intensity. Temperature, light intensity, and photoperiod was chosen to mimic natural spring conditions in the Wadden Sea (Rijkswaterstaat Waterinfo, 2023).

#### 2.4.2 Monitoring and data collection

**Figure 2:**
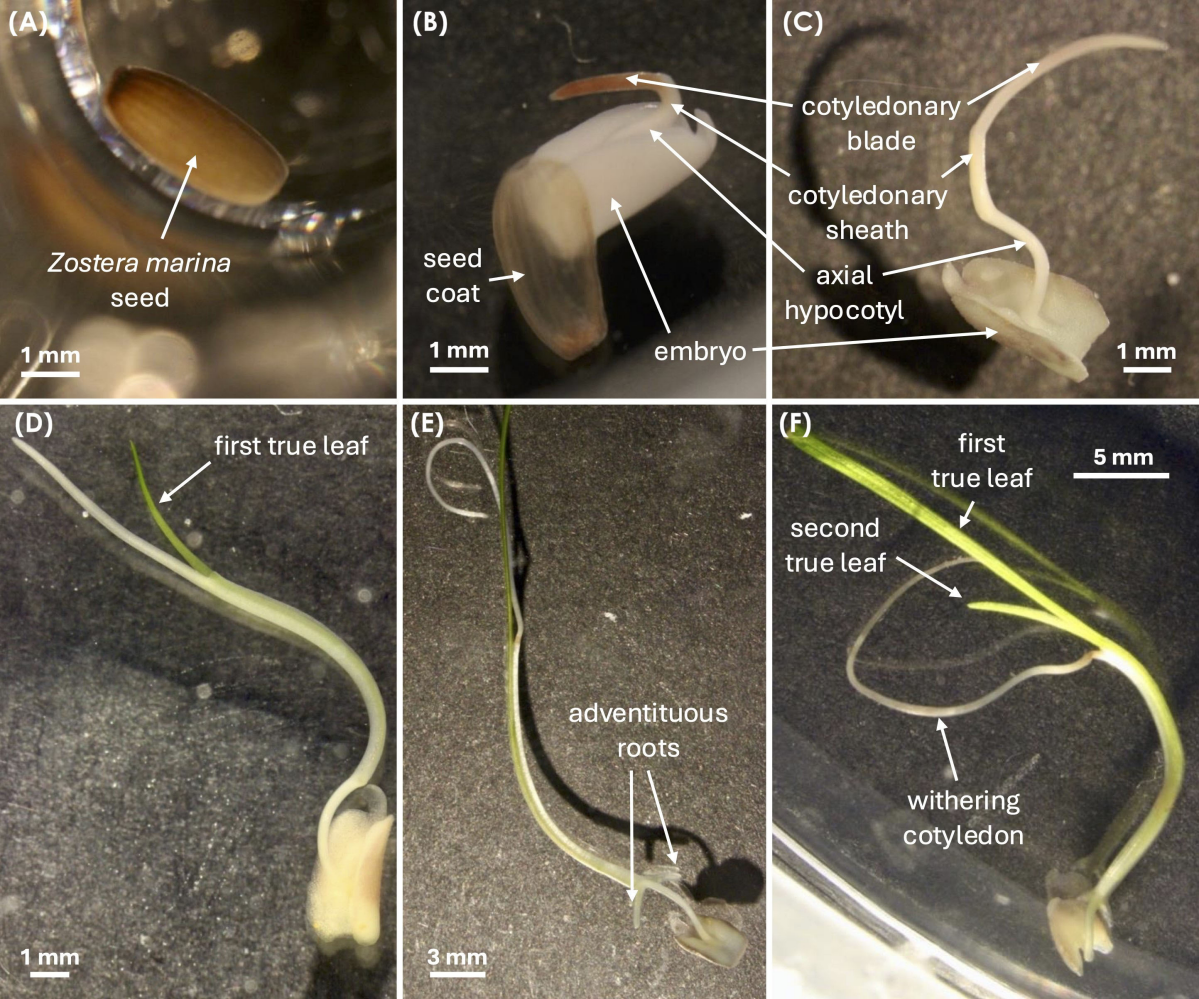
Developmental stages of *Zostera marina* seed germination and seedling growth, adapted from Xu et al. (2016). **(A)** Stage 0: Intact seed with seed coat. **(B)** Stage 1: Cotyledon emergence from the seed coat (germination). **(C)** Stage 2: Cotyledon elongation, showing the embryo, cotyledonary blade, cotyledonary sheath, and axial hypocotyl. **(D)** Stage 3: Emergence of the first true leaf. **(E)** Stage 4: Appearance of the first adventitious root. **(F)** Stage 5: Emergence of the second true leaf, followed by (Stage 6) cotyledon withering. All images were captured using a Leica MZ16 microscope with a Canon EOS 600D camera.

Seeds were monitored for 60 days, with observations conducted three times per week for the first three weeks and biweekly thereafter. Germinated seeds were transferred to 35 mm Petri dishes filled with 4 mL SSW, and photographed twice a week using a Leica MZ16 microscope coupled with a Canon EOS 600D camera, for morphometric measurement (**Figure 2**).

Cotyledon, first and second leaf, and root lengths were measured to the nearest 0.01 mm using ImageJ software (v.2.9.0) (Schneider et al., 2012) three times a week. The software was calibrated for pixel-μm conversion via a certified micrometer reticle (Pyser-SGI, serial n. SC1539). Measurements followed the natural curvature of the structures, ensuring accuracy. To minimize potential parallax errors, all specimens were positioned in a flat plane during imaging, and measurements were taken from standardized lateral or dorsal views. Morphological changes, mold formation, and seed mortality were recorded until the end of the experiment.

#### 2.4.3 Viability assessment

Prior to the start of the experiment, seeds were visually inspected; unripe, germinated, and/or broken seeds, as well as seeds with positive buoyancy in natural seawater, were discarded as suggested by Ruiz-Montoya et al. (2012) and Xu et al. (2019).

Upon experimental conclusion, seed viability was assessed on non-germinated seeds using a 2,3,5-Triphenyl-Tetrazoliumchloride (TTC) assay following the protocol described by Lakon (1949). Seeds were punctured with a syringe needle and immersed in 100 µL 1% TTC solution for 48 hours. Viable seeds are presented as the final percentage of viable seeds (Viability %) for each PGR treatment, concentration, and seed generation.

### 2.5 Statistical analysis

Results are presented as arithmetic mean ± binomial confidence interval, unless otherwise specified. All statistical analyses were conducted using the R statistical software (v.4.0.2; R Core Team, 2020).

Germination percentage (GP) and Mean Germination Time (MGT) were calculated using the R package *GerminaR* (Lozano-Isla et al., 2019). The TTC viability assessment was used to determine the actual number of viable seeds included in the experiment. This viability check allowed us to adjust our analyses and graphs based on the real number of viable seeds, ensuring more accurate results and interpretations.

The effects of strigolactones (SL) and smoke-water, each at ten different concentrations, on seed germination were analyzed using General Linear Mixed Models (GLM) implemented through the R package *stats* (Zuur et al., 2009). Backward stepwise selection based on the Akaike Information Criterion (AIC) was performed for model selection. Categorical predictor variables in the GLM included: seed generation, PGR treatment, PGR concentration. Model comparison was performed using ANOVA and Tukey’s Honest Significant Difference (HSD) test to assess the impact of variable exclusion. Exploratory analysis indicated that the random effect “experiment” did no contribute to the overall variability in seed germination and, therefore, for simplicity and parsimony, we opted for a simpler GLM model without random effects.

Time-to-event (survival) analysis was adopted using the R packages *survival* and *survminer* (Therneau and Grambsch, 2000) to examine the effects of the PGR treatments on the time to germination. Survival analysis evaluates both the occurrence of germination and the time required for germination, allowing for comparisons between treatments and concentrations (McNair et al., 2012). Variable selection for this analysis was guided by the outcomes of the GLM model’s ANOVA.

Effects of SL and smoke-water on seedling development were assessed based on post-germination measurements of cotyledon, first and second leaf, and primary and secondary root lengths on each seedling. Growth metrics were analyzed using Generalized Additive Models (GAM) via the *mgcv* package in RAM was chosen to evaluate the effects of seed generation and PGR treatment on continuous growth measurements, focusing on concentration-dependent trends and tissue-specific responses.

The final model included categorical variables: “tissue type”, “generation”, “treatment”, and “concentration”, with day modelled as a smooth variable. “Growth” was the response variable. Exploratory analysis indicated that the random effect “experiment” did no contributed to the overall variability in seedling development and, therefore, for simplicity and parsimony, we opted for a simpler GAM model without random effects for the post-germination phase. This decision was made to avoid overfitting and to focus on the primary fixed effects of interest while still maintaining the flexibility of smooth terms to capture potential nonlinear trends in the data. By using a GAM, we ensured a balance between model complexity and interpretability while sufficiently capturing the underlying trends in seedling development.

## 3 Results

### 3.1 Identification of biosynthesis and signalling genes in *Zostera marina*

Our analysis identified several orthologous genes in *Z. marina*, corresponding to key components of strigolactones (SL) and karrikins (KAR) pathways and supporting their applicability as plant growth regulators (PGRs) in our experimental treatments (**Table 1**).

**Table 1:** Identification of Zostera marina orthologs corresponding to key genes involved in strigolactone (SL), and smoke-water biosynthesis and signaling pathways.

Plant growth regulator	Context	Protein	*Arabidopsis thaliana* gene (locus)	*Arabidopsis thaliana* accession no.	*Zostera marina* gene ID
SL	Biosynthesis	D27	D27	At1g03055	NA
SL	Biosynthesis	MAX family	MAX1, MAX3, MAX4	At2g26170, At2g44990, At4g32810	ZOSMA_154G00480 and ZOSMA_31G00220, NA, ZOSMA_154G00070
SL	Biosynthesis	LBO	LBO	At3g21420	NA
SL	Signaling	MAX family	MAX2/ORE9	At2g42620	ZOSMA_117G00500
SL	Signaling	D14	D14	At3g03990	ZOSMA_234G00120
SL	Signaling	SMXL	SMXL6, SMXL7, SMXL8	At1g07200, At2g29970, At2g40130	ZOSMA_193G00250
KAR	Signaling	MAX family	MAX2/ORE9	At2g42620	ZOSMA_117G00500
KAR	Signaling	KAI2	KAI2/HTL	At4g37470	ZOSMA_53G00910
KAR	Signaling	SMAX1	SMAX1	At5g57710	ZOSMA_218G00150
KAR	Signaling	SMXL2	SMXL2	At4g30350	ZOSMA_218G00150

In terrestrial plants, strigolactone (SL) biosynthesis is initiated from carotenoid precursors through the sequential enzymatic activity of D27, CCD7 (MAX3), CCD8 (MAX4), and MAX1, which together produce bioactive SL molecules perceived by the D14 receptor (Waters et al., 2017). In parallel, karrikin (KAR) signaling involves the perception of smoke-derived butenolides, such as KAR_1_, by the KAI2 receptor. Both pathways share a common downstream signaling module that includes the F-box protein MAX2, which mediates the ubiquitination and degradation of SMXL repressors, thereby triggering developmental responses (Waters et al., 2013; Carbonnel et al., 2020).

In the *Z. marina* genome, orthologs of the key signaling genes D14, KAI2, MAX2, and SMXL were identified (**Table 1**), whereas canonical SL biosynthetic genes (D27, CCD7, CCD8, MAX1) appear to be absent or highly divergent, consistent with previous genomic analyses of seagrasses (Olsen et al., 2016; Ma et al., 2024).

### 3.2 Regulatory effects of PGRs on seed germination

**Figure 3:**
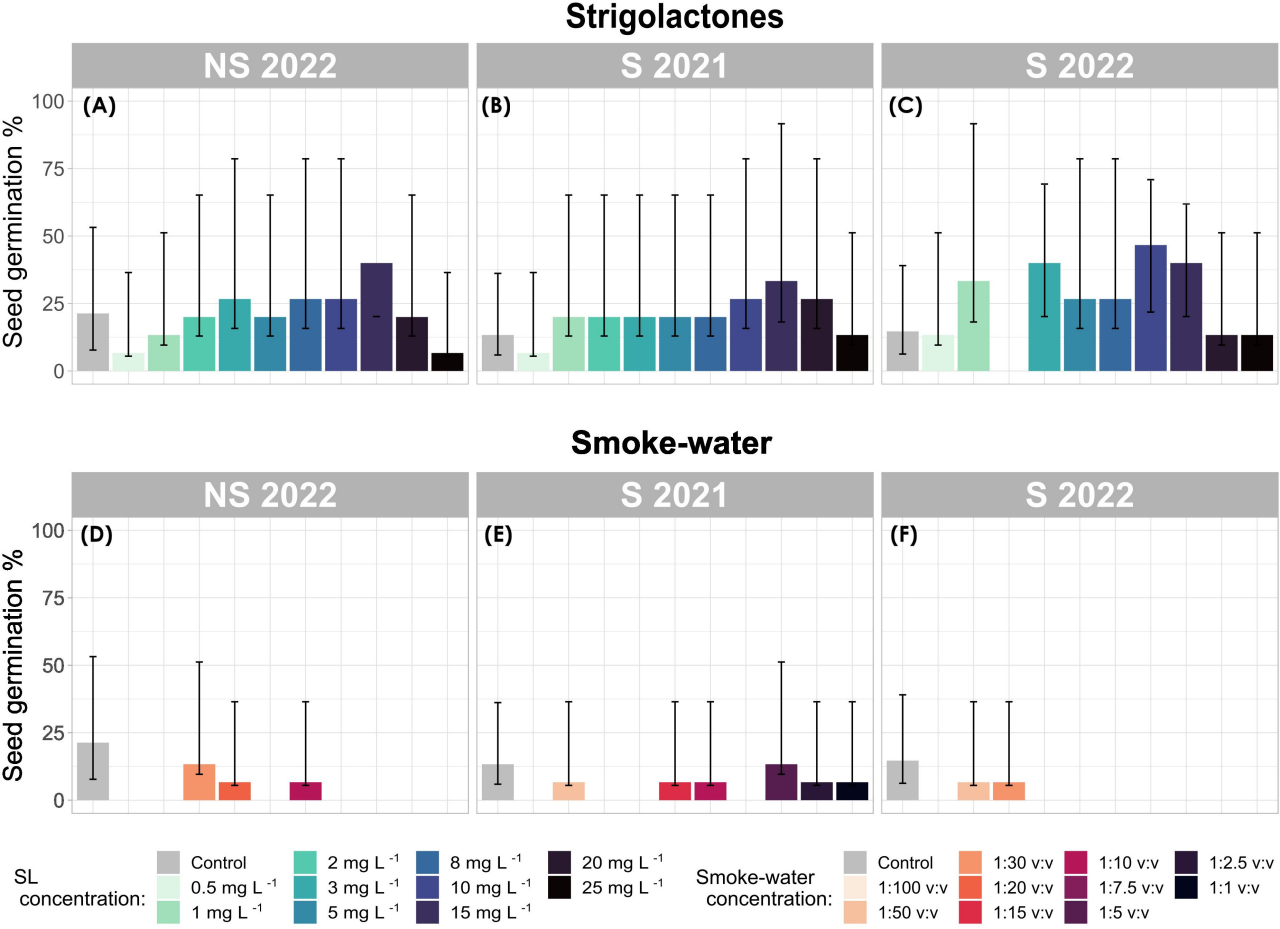
Seed germination (%) of *Zostera marina* across strigolactone (SL) **(A–C)** and smoke-water **(D–F)** treatments. Bar plots show mean germination percentage with binomial confidence intervals. Each panel represents a different seed generation (S 2021, S 2022, and NS 2022).

Logistic regression analysis revealed a significant effect of PGR treatments (χ²(2) 87.88, p = 2.2 e^-16^) on seed germination. Specifically, treatments with smoke-water had a significant inhibitory effect on seed germination (z = -2.307, p = 0.021), resulting in a lower overall germination percentage (GP) (2.9%, 95% CI: 12.2–21.8%) compared to the control group (16.44%, 95% CI: 12.2–21.8%) after 42 days of experimentation (**Figures 3D–F**). Although there were indications of a reduced inhibitory effect of smoke-water on older-generation seeds (S 2021) (**Figure 3E**), with a higher germination rate observed (GP: 4.7%, 95% CI 2.3–9.3%), this difference did not reach statistical significance.

In contrast, seeds treated with SL exhibited an overall positive germination response (**Figures 3A–C**), particularly in generation S 2022, where mean GP across all concentrations reached 25.3% (95% CI 19.1–32.8%), exceeding that of the control (GP: 14.7%, 95% CI 8.4–24.3%). High overall GP was also recorded in S 2021 (20.7%, 95% CI 14.9–27.8%, **Figure 3B**) and NS 2022 (20.7%, 95% CI 19.1–32.8%, (**Figure 3C**) under SL treatment. Seed generation did not appear to affect germination outcomes in response to SL (p = 0.61).

Germination success remained consistent across every SL concentration and, on average higher than control conditions (NS 2022 GP: 21.3%, 95% CI 13.6–31.9%; S 2022 GP: 14.6%, 95% CI 8.4–24.3%; and S 2021 GP: 13.3%, 95% CI 7.4–22.8%, **Figure 3**). However, significant increases in GP were only observed at SL concentrations of 15 mg L^-1^ (z = 2.92, p = 0.003), 10 mg L^-1^ (z = 2.53, p = 0.011), and 3 mg L^-1^ (z = 2.117, p = 0.034), with the highest GP occurring at these concentrations.

When considering the combined effects of seed generation, treatment, and concentration, the highest GP was recorded in S 2022 seeds treated with SL at 3 mg L^-1^ (40.0%, CI 95% 19.8–64.2%), 10 mg L^-1^ (40.0%, 95% CI 19.8–64.2%), and 15 mg L^-1^ (46.7%, 95% CI, 24.8–69.8%) (**Figure 3C**). However, no germination was recorded at 2 mg L^-1^ in S 2022.

### 3.3 Modulation of germination speed: contrasting effects of SL and smoke-water

**Figure 4:**
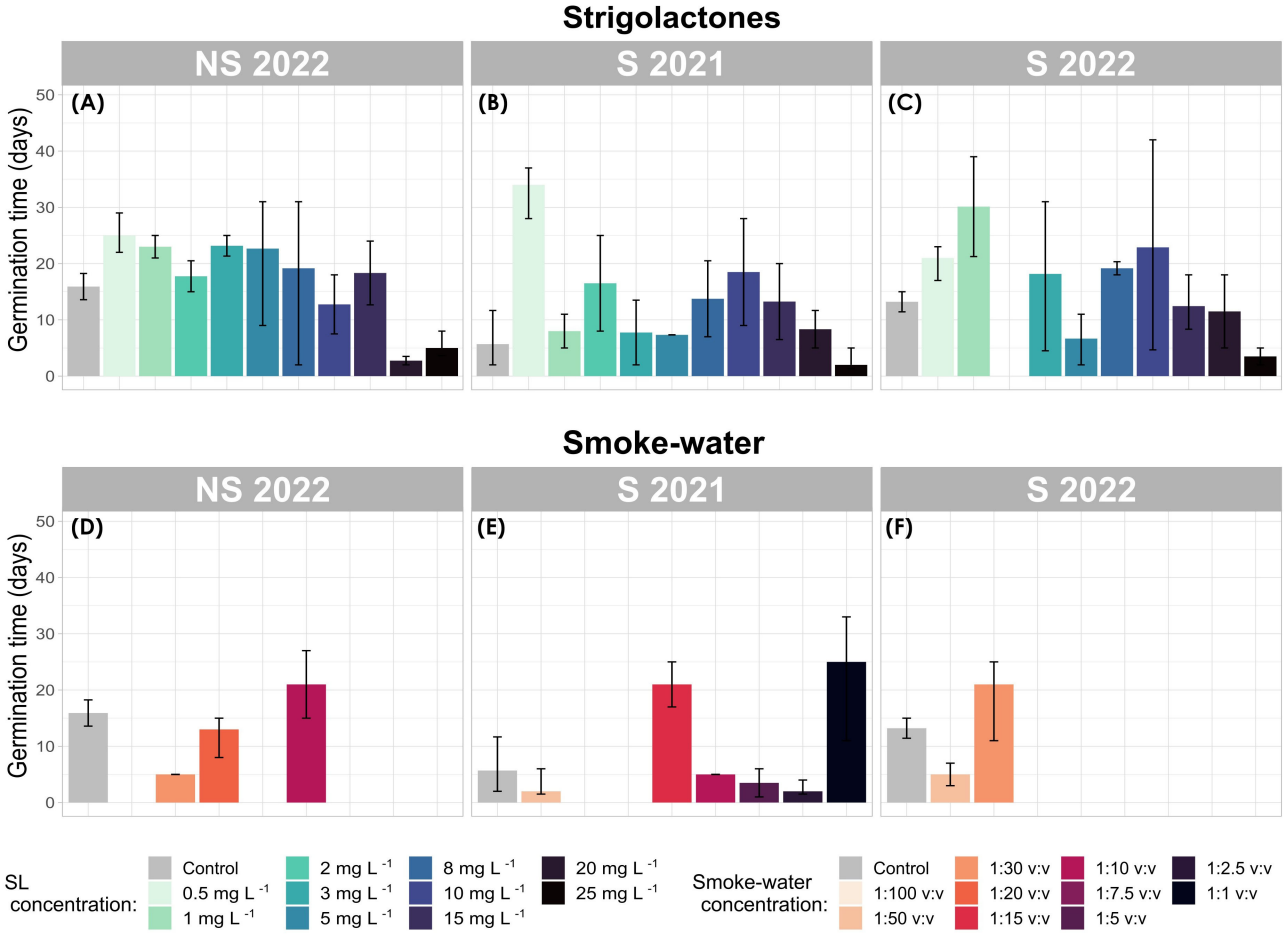
Mean germination time (MGT; days) of *Zostera marina* under strigolactone (SL) **(A–C)** and smoke-water **(D–F)** treatments. Bar plots represent mean values with confidence intervals. Each panel corresponds to a different seed generation (S 2021, S 2022, and NS 2022).

After a 42-day observation period, seeds from the control group exhibited the fastest mean germination time (MGT), averaging 10.76 ± 3.53 days (calculated across all seed generations, **Figure 4**). In contrast, seeds treated with SL had the slowest MGT, on average 15.23 ± 1.22 days (**Figures 4A–C**).

Seeds exposed to smoke-water exhibited a relatively low average MGT of 11.23 ± 2.82 days (**Figures 4D–F**). However, Cox regression analysis revealed that smoke-water treatments significantly reduced the hazard of germination by 89.64% compared to the control group (p = 0.03), indicating a strong inhibitory effect on germination. This aligns with previous observations of low germination rates across all smoke-water concentrations.

Median MGT values differed slightly, with SL-treated seeds at 15.12 ± 2.90 days and control seeds at 13.21 ± 2.33 days. Despite some S 2021 generation seeds treated with KAR at 1:50 v:v and 1:2.5 v:v concentrations exhibiting the fastest germination times across all treatments, these results were not statistically significant due to the low number of germinated seeds in these groups (**Figure 4E**).

SL treatment did not significantly influence MGT compared to the control (p = 0.40). Within SL treatments, the lowest MGT were observed at 20 mg L^−1^ and 25 mg L^−1^, but these differences were not statistically significant (p = 0.25 and p = 0.34, respectively). Cox regression analysis suggested that higher concentrations of SL increased the hazard of germination by an average of 1.90 times, potentially reducing MGT, although this effect was not statistically confirmed.

Notably, SL treatments at 3 mg L^−1^, 10 mg L^−1^, and 15 mg L^−1^ significantly accelerated germination (reduced MGT) over time. Germination occurred 2.7 times faster at 3 mg L^−1^ (p = 0.05), 3.1 times faster at 10 mg L^−1^ (p = 0.03), and 3.8 times faster at 15 mg L^−1^ (p = 0.008) as compared to the control.

Seeds from the S 2021 generation displayed the shortest MGT across all seed generations. The earliest MGT values were observed in the control treatment (5.69 ± 3.01 days) followed by smoke-water (9.75 ± 4.26 days), and SL (12.08 ± 2.24 days). Although these results were not statistically significant, Cox regression models indicated that seeds from S 2021 germinated 2.85 times earlier under smoke-water treatment and 1.62 times earlier under SL treatment than seeds from NS 2022 or S 2022.

### 3.4 Post-germination effects of SL and smoke-water on ontogenetic development in *Z. marina* seedlings

**Figure 5:**
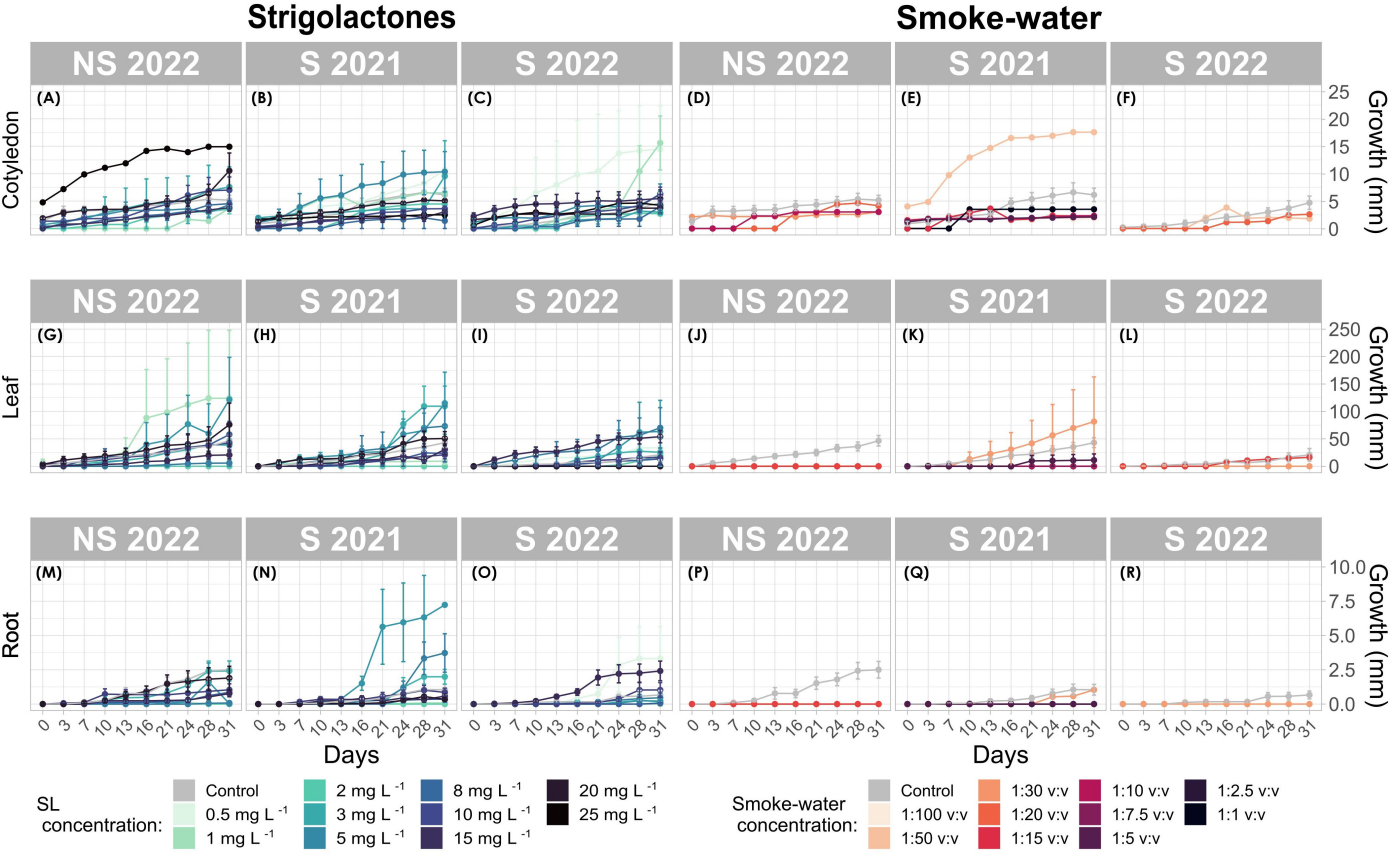
Seedling growth response over time for cotyledon, leaf, and root tissues of *Zostera marina*. **(A–C**, **G–I**, **L–N)** correspond to strigolactone (SL) treatments, while **(D-F**, **J-L**, **P-R)** correspond to smoke-water treatments. Each data point represents the mean tissue length (mm) with error bars indicating standard deviation.

SL treatments significantly promoted seedling growth, particularly at 10 mg L^-1^, which resulted in the most substantial increase in seedling biomass. At this concentration, cotyledon growth peaked at 238.2 mm in the S 2022 generation (**Figure 5C**), significantly outperforming that of control seedlings (p < 0.001). In contrast, higher SL concentrations (e.g. 25 mg L^-1^) led to growth suppression, particularly in root tissues (p < 0.001, **Figures 5M–O**), highlighting the concentration-dependent effects of SL on seedling development.

The interaction between SL and seed generation revealed that S 2022 seedlings exhibited the highest growth response at optimal SL concentrations (3 mg L^−1^, 10 mg L^−1^, and 15 mg L^−1^), with significant increases in cotyledon and root growth (p < 0.001) (**Figures 5C–O**). However, S 2021 seedlings showed a weaker growth response, with only marginal root development observed (Estimate = 5.99, p = 0.158, **Figure 5P**).

Smoke-water treatments generally exhibited a suppressive effect on seedling growth across all tissue types and concentrations tested. Lower smoke-water concentrations, such as 1 mg L^-1^, led to significant growth inhibition in both cotyledon and root tissues (Estimate = -15.15, p = 2.33e^-09^). S 2021 generation seedlings demonstrated a promotive response at low smoke-water concentrations (e.g. 1:50 v:v), with notable growth increases observed in root tissues (Estimate = 22.45, p = 1.73e^-11^). However, this positive interaction was not seen in the S 2022 generation, which exhibited reduced growth at the same concentration (Estimate = -18.12, p = 2e^-07^).

Cotyledon tissues were generally more sensitive to smoke-water, while root tissues displayed more variable responses. At higher smoke-water concentrations (e.g. 1:30 v:v), leaf growth in the S 2021 generation significantly improved (Estimate = 11.79, p = 0.0002), whereas the S 2022 generation showed no significant growth response at this concentration. However, given the limited number of germinated seeds under smoke-water treatment, these results may be incomplete or less conclusive.

SL treatments induced tissue-specific growth responses, primarily promoting cotyledon expansion rather than root or leaf development. For example, at 10 mg L^-1^, cotyledon growth was significantly enhanced (Estimate = -13.64, p = 0.0002), whereas root growth was suppressed at the same concentration, suggesting a selective stimulatory effect of SL on specific tissues.

Conversely, smoke-water treatments did not show strong tissue specificity, with growth inhibition observed uniformly across cotyledon, leaf, and root tissues.

Overall, SL-treated seedlings exhibited the highest levels of development compared to other treatments and the control. S 2022 seedlings treated with 2 mg L^-1^ SL displayed the highest cotyledon growth (23.8 mm), followed closely by those treated with 0.5 mg L^-1^ SL (22.4 mm). These values significantly exceeded those recorded in both control and smoke-water-treated seedlings. In contrast, seedlings exposed to high smoke-water concentrations exhibited markedly reduced growth, with root lengths as low as 0.169 mm in NS 2022 seedlings (**Figure 5**).

### 3.5 Seed viability

Post-germination viability assessments were performed on non-germinated seeds using 2,3,5-Triphenyl-Tetrazoliumchloride (TTC) staining to determine embryonic viability. The results revealed a high viability rate, with 97.5% to 99.5% of seeds staining positively (**Supplementary Table 1**).

## 4 Discussion

Our results provide the first experimental evidence of strigolactones (SL) and smoke-water effects on seed germination in *Zostera marina*, revealing notable differences in the effect of these two plant growth regulators at different concentrations. SL exhibited have a stimulatory effect on germination under specific conditions, while smoke-water consistently inhibit germination. These findings align with the broader understanding of phytohormonal regulation of seed germination.

### 4.1 Effect of SL on germination and seedling development

The role of SL in terrestrial plant species, particularly on seed germination and development, is relatively well-established (Al-Babili and Bouwmeester, 2015; Waters et al., 2017; Trasoletti et al., 2022; Seto, 2023), yet their function in aquatic ecosystems remains largely unexplored. Aquatic plants, especially in marine and estuarine ecosystems, are exposed to highly variable environmental conditions, including fluctuations in light availability, nutrients, temperature, and salinity, which may influence SL-mediated regulation of seed germination differently compared to their terrestrial ancestors.

Recent studies have identified key pathways potentially associated with SL signaling in marine plant species, such as *Z. marina* (Olsen et al., 2016; Ma et al., 2024). Our results support the presence of a functional SL pathway in seagrass, although its specific role in their germination processes remains to be elucidated. The significant promotive effect of SL on *Z. marina* seed germination observed in this study are consistent with findings from terrestrial systems, where SLs stimulate germination in both parasitic and non-parasitic species (Wang et al., 2010; Delaux et al., 2012; Strullu-Derrien et al., 2018; Poveda, 2024; Vernié et al., 2025). The concentrations used in this study align with those reported to induce germination in model parasitic taxa such as *Striga* and *Orobanche* (Pouvreau et al., 2013; Chen et al., 2021), and fall within the physiological range known to activate SL-mediated responses in other plant species. Concentrations as high as 20 μM (~ 6 mg L^-1^) have been shown to effectively trigger germination in species from these genera, while higher levels have been applied to mitigate abiotic stress, including drought and salinity, in crops such as wheat and alfalfa, where GR24 has been observed to modulate both physiological and biochemical responses (Ling et al., 2020; Yang et al., 2023).

In terrestrial systems, SL are key signaling molecules that regulate germination for SL-responsive species in response to environmental cues, particularly under nutrient-limited conditions (Akiyama et al., 2005; Brun et al., 2018). SL also serve as host-derived signals that facilitate symbiotic interactions with arbuscular mycorrhizal fungi (AMF) (Ruyter-Spira and Bouwmeester, 2012; Brewer et al., 2013; Mitra et al., 2021). SL can also play a role in parasitic relationships, triggering the germination of root parasites (e.g. *Striga* and *Orobanche* spp.), underscoring their broader significance as regulators of plant–microbe and plant–plant interactions (Cook et al., 1966; Matusova et al., 2005). However, there is currently no evidence of the formation of arbuscular mycorrhizal symbiosis (AMS) in seagrass species, including *Z. marina* (Ma et al., 2024).

Interestingly, *Z. marina* has retained AMS-conserved genes, such as *MAX2* (**Table 1**), which may serve non-symbiotic roles related to SL signaling. The retention of these genes suggests that SL may have evolved distinct functions in seagrasses (**Table 1**; Olsen et al., 2016; Waters et al., 2017; Ma et al., 2024), possibly linked to seed germination, root development, and responses to abiotic stress. In seagrasses and other aquatic angiosperms, SL-related genes have been proposed to participate in light perception, salinity tolerance, and hormone crosstalk under submerged conditions (Olsen et al., 2016; Shen et al., 2022; Ma et al., 2024).

Furthermore, the positive effect of SL on germination was consistent across seed generations, as seen in the germination response observed across multiple seed cohorts. This suggests that SL-mediated germination is not generation-dependent but rather a stable response to SL treatment. These findings align with the role of SL in parasitic plants, where these molecules act as germination cues for long-dormant seeds (Nelson, 2021).

In terrestrial species, such as *Arabidopsis thaliana* and *Oryza sativa*, SL often works in concert with abscisic acid (ABA) and gibberellins (GA), which are also operational in marine plants, to regulate germination, development, and stress responses (Sabagh et al., 2021; Sharma et al., 2024). Whether similar interactions exist in marine species remains uncertain, but our results suggest that SL may play broader roles in *Z. marina*, potentially extending beyond their established functions in terrestrial species.

The delayed mean germination time (MGT) observed under higher-concentration SL treatments potentially indicate that SL may also influence the timing of germination, possibly through interactions with ABA and GA. In species such as *Orobanche minor*, SL are known to antagonize ABA during seed conditioning, with full dormancy release and germination triggered by exposure to the synthetic SL analog GR24 (Cheng et al., 2013; Jamil et al., 2014). Although direct SL–ABA functional interactions have not yet been demonstrated in marine angiosperms or other aquatic plants, the conserved signaling architecture and ecological context support the hypothesis that SL modulate, at least partially, ABA-mediated inhibition of germination in *Z. marina*. Thus, it is plausible that similar regulatory mechanisms exist in *Z. marina*, where SL might regulate responses to shallow, submerged conditions, including sensitivity to fluctuations in light availability and temperature, typical of coastal environment.

The lack of evidence for AMS in *Z. marina* (Ma et al., 2024), further supports the hypothesis that SL have evolved distinct roles in marine plants. The retention of AMS-conserved genes, such as *MAX2* in *Z. marina* (Olsen et al., 2016), suggests a shift in SL function, potentially toward regulating processes independent of microbial symbiosis. While the number of MAX2 copies in *Z. marina* remains unclear, further analysis would be required to determine whether gene duplication has led to functional diversification. This secondary loss of AMS-specific genes implies that SL in seagrasses may have adapted to regulatory roles related to the optimization of the germination process in response to environmental inputs (exogenous), such as nutrient availability, stress tolerance, or developmental timing in marine environments. Furthermore, the consistent germination outcomes across seed generations and SL treatments, coupled with the slower MGT, suggest that SL may help mediate adaptive responses to submerged conditions in shallow water by regulating both the initiation and timing of germination.

Recent studies have demonstrated that the synthetic strigolactone analog GR24 stimulates rhizoid growth in bryophytes such as the moss *Physcomitrella patens* and the liverwort *Marchantia* species, as well as in the charophyte *Chara corallina* (Delaux et al., 2012). The presence of *D14*-like genes, which encode α/β-hydrolase receptors specific for SL, in charophytes, the closest relatives of land plants, marks the earliest conservation of SL signaling components in algae (Domozych et al., 2017). In *Z. marina*, we identified SL signaling orthologs, including *D14*-like and F-box (*MAX2*-like) proteins (**Table 1**), consistent with previous reports of their presence in the *Z. marina* genome (Olsen et al., 2016; Ma et al., 2024). These findings raise questions regarding the evolutionary conservation and potential diversification of SL function in seagrasses. Interestingly, the *D14* gene shows high conservation in later-evolving plant lineages, such as gymnosperms and angiosperms (Ruyter-Spira and Bouwmeester, 2012).

Our findings demonstrate that SL positively affects *Z. marina* seed germination (**Figure 3**) and seedling development (**Figure 5**), supporting a functional role in this marine angiosperm. This sensitivity to SL may reflect an ancestral signaling role retained from terrestrial ancestors or represent a novel function independent of AMF interactions. Building on this perspective, the evolutionary emergence of SL function in freshwater algae may be linked to their gradual shift toward moist terrestrial habitats (Becker and Marin, 2009). This environmental transition likely exerted selective pressure favoring rhizoid development for enhanced anchorage and facilitation of water and nutrient uptake. The stimulation of rhizoid elongation by SL aligns with known SL effects, such as promoting protonema expansion in mosses (Proust et al., 2011), root hair elongation in angiosperms (Jan et al., 2024) and inducing hyphal growth and branching in AMS (Besserer et al., 2006).

Our results further suggest that SL treatments in *Z. marina* induce tissue-specific responses, enhancing expansion of the cotyledon more than that of root or leaf tissues. While cotyledon growth was stimulated across SL concentrations, root growth was suppressed at higher SL levels but promoted at low and mid concentrations. This suggests selective effects on different tissues and implies a conserved physiological role of SL in promoting structures essential for light perception, nutrient acquisition, and anchorage.

### 4.2 Inhibitory effects of smoke-water on germination

Smoke-water comprises a group of butenolide molecules (e.g. KAR_1_) produced by the combustion of plant-derived materials and is known to induce seed germination in fire-follower terrestrial plant species (Flematti et al., 2004). Unlike SL, which are endogenous plant hormones, smoke-water compounds are not known to be synthesized by plants but are instead perceived through the KAI2 receptor, which has homology with the SL receptor D14 (Waters et al., 2014). The role of smoke-water compounds in plant development extends beyond fire-related germination responses, with evidence suggesting broader physiological functions, including root development, stress responses, and adaptation to environmental cues (Nelson et al., 2012; Waters et al., 2014).

Our study reveals that, in contrast to SL, smoke-water exhibit a significant inhibitory effect on the germination of *Zostera marina* seeds across all tested concentrations and seed generations. These findings support the hypothesis that smoke-water can act as a germination inhibitor, potentially serving as an adaptive mechanism to prevent premature germination under suboptimal environmental conditions (Nelson et al., 2012), as observed in fire-adapted Mediterranean genera, typical of coastal environments (e.g. *Pinus* etc).

Interestingly, a slight reduction in the inhibitory effect of smoke-water on seed germination was observed in the older generation seeds (S 2021). Germination inhibition remained pronounced across seed generations, with older seed generations displaying reduced sensitivity to smoke-water. This observation aligns with findings in terrestrial fire-follower species, where prolonged seed dormancy is prevalent and germination is often stimulated by fire-related cues, such as in *Pinus halepensis* (Waters et al., 2013), *Cistus salvifolius* (Çatav et al., 2018), *Banksia attenuata* (Jefferson et al., 2014), and *Eucalyptus globulus* (Chiwocha et al., 2009). However, the broader ecological significance of smoke-water sensitivity across diverse plant lineages, including marine species, remains poorly understood (Chiwocha et al., 2009). Nelson et al. (2009) proposed that active compounds present in smoke-water, particularly butenolides such as karrikinolide (KAR_1_) and related karrikin-like molecules, may be produced through mechanisms other than fire. These compounds could therefore have an endogenous signaling role, potentially influencing processes such as seed dormancy release, germination timing, and early seedling development. However, whether these compounds present serves distinct ecological functions in non-fire-adapted species or if their effects arise as a byproduct of structural similarity and receptor recognition remains unclear. Further research is needed to determine whether mole are produced in other ecological contexts and whether they interact with unidentified endogenous compounds relevant to plant development.

As summarized in **Table 1**, the identification of *Arabidopsis thaliana* mutants insensitive to karrikins (KAR) has been instrumental in uncovering key components of the KAR signaling. Two essential genes were identified: *MORE AXILLARY GROWTH2* (*MAX2*), previously seen also for its role in SL signaling, and *KARRIKIN-INSENSITIVE2* (*KAI2*), which shares significant homology with the SL receptor gene *DWARF14* (*D14*) (Smith, 2014; Waters et al., 2014).

Although it was hypothesized that KAR may mimic SL due to their shared butenolide ring structure, evidence indicates that in *A. thaliana*, KAR and SL are perceived by paralogous receptors that evolved from a gene duplication event. While *KAI2* and *D14* retain structural similarities, they have functionally diverged to mediate distinct physiological responses through separate signaling pathways (Nelson et al., 2009). Our *in silico* analysis confirms the presence of orthologs of *MAX2*, *D14*, and *KAI2* in the *Z. marina* genome (**Table 1**), suggesting that similar signaling pathways may exist in this marine species.

Despite the structural similarities between KAR and SL, there is no evidence that plants synthesize KAR themselves, indicating that the endogenous compound interacting with *KAI2* is likely analogs to SL, given the similarity between *KAI2* and *D14* (Waters et al., 2014).

Our results also support the potential role of active compounds present in smoke-water in root development, specifically by suppressing root elongation. Similar outcomes have been reported for *Lotus japonicus*, where KAR treatments led to shorter primary roots and elongated root hairs (Carbonnel et al., 2020). Additionally, studies have shown that KAR enhances early growth in several plant species by modulating enzymatic cascades involved in essential cellular activities, including glycolysis, redox homeostasis, and secondary metabolism (Li et al., 2018; Rehman et al., 2018; Wang et al., 2020).

In terrestrial plant species, KAR have been suggested to modulate physiological and biochemical processes to counteract the detrimental effect of salt and drought stress (Jamil et al., 2014; Malook et al., 2014; Shah et al., 2020). *Z. marina* thrives in dynamic marine environments, where it faces abiotic stress due to this dynamism, such as freshwater input, temperature fluctuations, and high turbidity following heavy rains and storms (Nejrup and Pedersen, 2008). If *KAI2* contributes to modulating physiological responses to environmental pressures, it may play a role in adaptation to these challenges.

Additionally, factors like turbidity significantly reduce light availability in marine environment, leading to decreased photosynthetic efficiency and impaired growth in marine plants. Similarly, in terrestrial ecosystems, low red to far-red light ratios and shade stress reduce plant productivity, resulting in reduced seed germination and seedling growth (Casal, 2013; de Wit et al., 2016). In *A. thaliana*, exposure to KAR has been shown to enhance tolerance to low-light conditions by increasing chlorophyll content, increasing photosynthetic capacity, and promoting hypocotyl elongation (Nelson et al., 2010).

Similar to SL signaling with *D14* and *MAX2* genes, the conservation of the *KAI2* gene across plant lineages (and also found in algae and bacteria) suggests an ancestral or fundamental function (Waters et al., 2013). Supporting this, experiments have shown that introducing KAI2 from a non-seed plant species (e.g. *Selaginella*, which does not respond to KAR or SL) into an *A. thaliana* mutant lacking KAI2 successfully restored seedling development (Smith and Li, 2014), hence potentially another function than SL and KAR response is to be sought. On the other hand, Flematti et al. (2015) observed that *A. thaliana* mutants lacking *KAI2* exhibited increased seed dormancy, elongated hypocotyls, and narrow leaves. Similar phenotypes have also been witnessed in other studies and species (Scaffidi et al., 2014; Swarbreck et al., 2019, 2020; Villaécija-Aguilar et al., 2019; Wang et al., 2020). In our study, some smoke-water-treated seedlings exhibited comparable phenotypic responses, including delayed cotyledon emergence, reduced cotyledon elongation, and limited seedling growth, suggesting that *KAI2* may play a functional role in *Z. marina*.

The observed inhibitory effect of smoke-water on *Z. marina* seeds implies a possible adaptation in KAI2 function within this species. This adaptation may be especially relevant in the aftermath of terrestrial fires, where rainfall carries ash and debris (rich in butolenide compounds) into rivers and coastal lagoons, thereby elevating turbidity and creating suboptimal germination conditions and effectively creating smoke-water solution. Under these circumstances, the interplay between active compounds present in smoke-water and KAI2 could serve as an adaptive mechanism, allowing *Z. marina* to postpone germination and seedling development until environmental conditions improve. This regulatory process may enhance the species’ survival and resilience under challenging conditions, thereby contributing to its persistence in dynamic coastal ecosystems.

In conclusion, our findings suggest that active compounds present in smoke-water plays a multifaceted role in regulating seed germination and plant development in *Z. marina*. The presence of *MAX2*, *D14*, and *KAI2* orthologs indicates that KAR and SL pathways are evolutionarily conserved in this marine species. The inhibitory effects of smoke water on germination and root elongation, along with the potential modulation of *KAI2* function, highlight the complex interplay between environmental signals and plant developmental processes. Further research is needed to elucidate the endogenous compounds interacting with *KAI2* in *Z. marina* and to understand how these signaling pathways contribute to the plant’s adaptation to its marine shallow subtidal habitat.

### 4.3 Implications, limitations and future research

Our findings provide new insights into the role of SL and smoke-water in *Zostera marina* seed germination, suggesting that while these regulators have conserved functions, their roles in marine plants may differ from those observed in terrestrial species. This expands our understanding of SL- and KAR-mediated processes beyond their traditional roles in land plants, contributing to the broader field of marine plant biology. While the application of PGRs holds potential for enhancing and optimizing seed germination and seedling establishment in marine plant restoration, this study provides preliminary insights into the physiological responses of *Z. marina* to strigolactone (SL) and smoke-water (a source of karrikin activity) treatments.

As with many experimental studies, there are limitations to our approach. The precise mechanisms by which SL and KAR interact with other hormones, such as ABA and GA, remain unclear in marine plants, and further research is needed to determine whether similar regulatory networks exist. Our experimental design addressed potential biases commonly encountered in studies of this nature. For instance, we implemented a non-sterilized control (NS 2022) combined with a full set of SL and smoke-water concentrations to minimize the potential effect of sterilizing agents. Additionally, we repeated the experiments three times to account for variability in seed maturation and time effect, and we used independent replicates within each treatment to ensure robustness. Furthermore, synthetic KAR was not commercially available in Europe at the time of our experiments, requiring us to use self-synthesized smoke-water solutions.

Moreover, although the selected concentrations for SL and the protocol for KAR are commonly used in terrestrial plant research, particularly in studies involving root-parasitic plants, extreme environmental stressors, and post-fire mechanisms for population resilience, their application in marine plants remains unexplored. Hence, we adopted a wide gradient of SL and KAR concentrations, allowing us to explore both promotive and inhibitory effects of SL and gain a comprehensive understanding of dose-dependent responses.

While we observed significant effects of SL and smoke-water on germination, their roles in later developmental stages remain poorly understood, particularly regarding the impact of SL and smoke-water application during seedling establishment.

Future research should focus on elucidating the molecular pathways underlying SL and KAR signaling in *Z. marina* and seagrasses more broadly, as well as investigating potential interactions with other hormonal pathways. Expanding these studies to other marine angiosperms will help determine whether these findings are broadly applicable across aquatic plants.

## Data Availability

The raw data supporting the conclusions of this article will be made available by the authors, without undue reservation.
