# Supplementary material for: Unlocking seagrass germination: divergent roles of strigolactones and smoke-water in Zostera marina (Zosteraceae)
Source: Front Plant Sci. 2025 Nov 18;16:1629832. doi: 10.3389/fpls.2025.1629832 (PMC12670585; doi:10.3389/fpls.2025.1629832)
Supplement: Supplementary file 1 [file Table1.docx]

| **Generation** | **Hormone** | **Concentration** | **Seed Viability (%)** |
| --- | --- | --- | --- |
| NS 2022 | Control | Control | 98.5 |
| NS 2022 | KAR | 1:1000 v:v | 98.2 |
| NS 2022 | KAR | 1:50 v:v | 99.1 |
| NS 2022 | KAR | 1:30 v:v | 97.5 |
| NS 2022 | KAR | 1:20 v:v | 98.7 |
| NS 2022 | KAR | 1:15 v:v | 99.3 |
| NS 2022 | KAR | 1:10 v:v | 97.8 |
| NS 2022 | KAR | 1:7.5 v:v | 98.5 |
| NS 2022 | KAR | 1:5 v:v | 99 |
| NS 2022 | KAR | 1:2.5 v:v | 97.9 |
| NS 2022 | KAR | 1:1 v:v | 98.6 |
| NS 2022 | SL | 0.5 mg L^-1^ | 97.7 |
| NS 2022 | SL | 1 mg L^-1^ | 98.9 |
| NS 2022 | SL | 2 mg L^-1^ | 99.2 |
| NS 2022 | SL | 3 mg L^-1^ | 97.6 |
| NS 2022 | SL | 5 mg L^-1^ | 98.3 |
| NS 2022 | SL | 8 mg L^-1^ | 99.4 |
| NS 2022 | SL | 10 mg L^-1^ | 98.1 |
| NS 2022 | SL | 15 mg L^-1^ | 97.9 |
| NS 2022 | SL | 20 mg L^-1^ | 98.7 |
| NS 2022 | SL | 25 mg L^-1^ | 99 |
| S 2022 | Control | Control | 99 |
| S 2022 | KAR | 1:1000 v:v | 98.4 |
| S 2022 | KAR | 1:50 v:v | 99.1 |
| S 2022 | KAR | 1:30 v:v | 97.6 |
| S 2022 | KAR | 1:20 v:v | 98.8 |
| S 2022 | KAR | 1:15 v:v | 99.5 |
| S 2022 | KAR | 1:10 v:v | 97.7 |
| S 2022 | KAR | 1:7.5 v:v | 98.6 |
| S 2022 | KAR | 1:5 v:v | 98.2 |
| S 2022 | KAR | 1:2.5 v:v | 99.3 |
| S 2022 | KAR | 1:1 v:v | 97.8 |
| S 2022 | SL | 0.5 mg L^-1^ | 97.9 |
| S 2022 | SL | 1 mg L^-1^ | 98.6 |
| S 2022 | SL | 2 mg L^-1^ | 99.4 |
| S 2022 | SL | 3 mg L^-1^ | 98.1 |
| S 2022 | SL | 5 mg L^-1^ | 97.5 |
| S 2022 | SL | 8 mg L^-1^ | 98.9 |
| S 2022 | SL | 10 mg L^-1^ | 99.2 |
| S 2022 | SL | 15 mg L^-1^ | 97.8 |
| S 2022 | SL | 20 mg L^-1^ | 98.5 |
| S 2022 | SL | 25 mg L^-1^ | 99 |
| S 2021 | Control | Control | 97.8 |
| S 2021 | KAR | 1:1000 v:v | 97.8 |
| S 2021 | KAR | 1:50 v:v | 99.3 |
| S 2021 | KAR | 1:30 v:v | 98.2 |
| S 2021 | KAR | 1:20 v:v | 97.6 |
| S 2021 | KAR | 1:15 v:v | 99.1 |
| S 2021 | KAR | 1:10 v:v | 98.7 |
| S 2021 | KAR | 1:7.5 v:v | 99 |
| S 2021 | KAR | 1:5 v:v | 97.9 |
| S 2021 | KAR | 1:2.5 v:v | 98.4 |
| S 2021 | KAR | 1:1 v:v | 99.2 |
| S 2021 | SL | 0.5 mg L^-1^ | 98.1 |
| S 2021 | SL | 1 mg L^-1^ | 99.4 |
| S 2021 | SL | 2 mg L^-1^ | 97.9 |
| S 2021 | SL | 3 mg L^-1^ | 98.6 |
| S 2021 | SL | 5 mg L^-1^ | 99 |
| S 2021 | SL | 8 mg L^-1^ | 97.5 |
| S 2021 | SL | 10 mg L^-1^ | 98.3 |
| S 2021 | SL | 15 mg L^-1^ | 99.2 |
| S 2021 | SL | 20 mg L^-1^ | 97.7 |
| S 2021 | SL | 25 mg L^-1^ | 98.9 |
